# Multiple Cutaneous Squamous Cell Carcinomas on Primary Epidermodysplasia Verruciformis in a 19-Year-Old Black Subject

**DOI:** 10.1155/crdm/9936485

**Published:** 2025-11-09

**Authors:** Panawé Kassang, Abas Mouhari-Toure, Toukilnan Djiwa, Sefako Abla Akakpo, Julienne Noudé Teclessou, Bayaki Saka, Palokinam Pitche

**Affiliations:** ^1^Department of Dermatology and Venerology of CHU Kara, University of Kara, Kara, Togo; ^2^Departement of Pathologic Anatomy and Cytology of CHU Kara, University of Kara, Kara, Togo; ^3^Departement of Dermatology and Venerology of CHU Sylvanus Olympio, University of Lomé, Lomé, Togo; ^4^Departement of Dermatology and Venerology of CHU Campus, University of Lomé, Lomé, Togo; ^5^Skin and Environment Research Laboratory, University of Lomé, Lomé, Togo

**Keywords:** Africa, Black, cutaneous carcinomas, epidermodysplasia verruciformis

## Abstract

**Introduction:**

Epidermodysplasia verruciformis (EV) is a rare genodermatosis characterized by chronic human papillomavirus infection of the skin and a risk of carcinomatous degeneration. We report a case of EV complicated by multiple cutaneous carcinomas of the scalp in a 19-year-old subject.

**Case Presentation:**

A 19-year-old male, phototype VI according to Fitzpatrick's classification, was referred to the dermatology department of CHU Kara with multiple painful tumoral lesions of the scalp that had been evolving for about 1 year. Clinical examination revealed hypochromic macules (Pityriasis versicolor-like), hypochromic verrucous flat papules scattered over the face, scalp, neck, trunk, and upper limbs. These lesions were associated with occasional pruritus. Apart from these lesions, there were three ulcerative-bourgeonous tumors on the scalp. A normal blood count was obtained, and the HIV serological test was negative. Histological examination of lesion biopsies confirmed the diagnosis of squamous cell carcinoma for all three lesions. Patient management was limited by lack of financial resources.

**Conclusion:**

As sub-Saharan Africa is characterized by fragile health systems and high solar gradients, particular emphasis must be placed on preventive measures for skin cancers in patients with genodermatoses at risk of degeneration.

## 1. Introduction

Epidermodysplasia verruciformis (EV) is a rare genodermatosis characterized by abnormal susceptibility to chronic human papillomavirus (HPV) infection of the skin [1]. This mostly autosomal recessive condition due to mutation of the EVER1 and EVER2 genes is characterized by flat verrucous lesions and Pityriasis versicolor-like hypochromic macules [2]. These lesions are often induced by oncogenic HPV strains and, in some cases, develop into skin cancers, notably cutaneous squamous cell carcinomas [2, 3]. There are also acquired forms associated with immunocompromised conditions [1]. Acquired forms associated with HIV are most common in sub-Saharan Africa [4, 5]. In our study, we report a case of primary EV in a sub-Saharan immunocompetent subject, complicated by multiple squamous cell carcinomas.

## 2. Case Presentation

A 19-year-old male, phototype VI according to the Fitzpatrick classification, was referred to the dermatology department of CHU Kara for multiple painful tumoral lesions of the scalp that had been evolving for about 1 year. The patient had no known general pathological antecedents except for a progressively extensive dermatosis of the photoexposed areas of the face, trunk and limbs, which he had had since the age of 13 and for which he had never consulted a dermatologist. His biological parents were not consanguineous, and none of his siblings had the same dermatosis.

**Figure 1:**
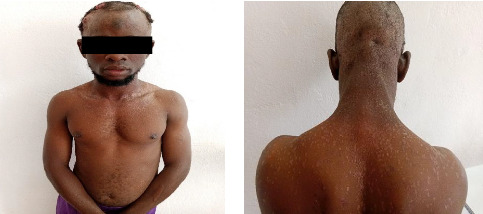
Pityriasis versicolor-like skin lesions of the trunk neck and head. (a) Front view. (b) Back view.

**Figure 2:**
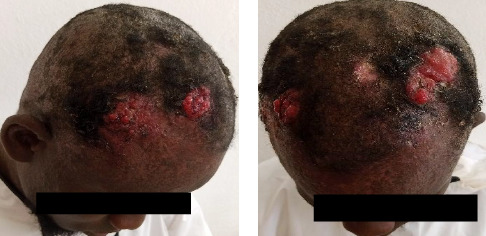
Squamous cell carcinoma tumors of the scalp. (a) Right view. (b) Front view and left view.

Clinical examination revealed hypochromic macules (Pityriasis versicolor-like) and hypochromic verrucous papules on the face, scalp, neck, trunk, and upper limbs (Figures 1(a) and 1(b)). These lesions were associated with occasional pruritus. Apart from these lesions, there were three ulcerative and exophytic tumors on the scalp. The first tumor was located in the right temporal region and measured 4 × 6 cm (Figure 2(a)). The second tumor was located in the right frontal region and measured 4 × 3 cm (Figure 2(b)). The third tumor was located in the left fronto-parietal region and measured 7 × 3 cm (Figure 2(b)). These lesions were painful and bled on contact. Examination of the various lymph nodes in the head and neck revealed no adenopathy, and the rest of the clinical examination was normal.

**Figure 3:**
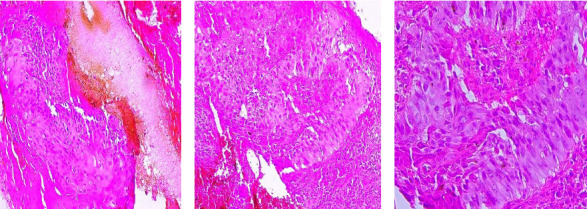
Histological appearance of squamous cell carcinoma. (a) Hematoxylin and eosin (HE) ×40. (b) HE ×100. (c) HE ×400.

A blood count was normal, and HIV serology was negative. Histological examination of lesion biopsies confirmed the diagnosis of squamous cell carcinoma for all three lesions (Figures 3(a), 3(b) and 3(c)). Histological lesions were described as carcinomatous proliferation consisting of moderately differentiated squamous cell lobules within a fibro-inflammatory and hemorrhagic stroma invading the entire epidermis and dermis (Figure 3(a)). We requested a craniofacial tomodensitometry, which could not be carried out due to lack of funds. As the surgical approach in this patient was complex, we proposed treatment with general anticancer drugs, which the patient was also unable to purchase for financial reasons. The patient is currently undergoing symptomatic treatment while awaiting the necessary funds.

## 3. Discussion

EV is one of the rarest genodermatoses in the literature. Only around 500 cases have been reported worldwide by Imarhorn et al. in a global review of the literature in 2017 [6]. Although EV is considered a genetic disease, research has led to a better understanding of viral infection and its role in carcinogenesis. A distinction is made between two forms: the classical, hereditary, autosomal recessive form and the acquired, autosomal recessive form. The second is the acquired form, which is secondary and clinically almost indistinguishable from the first. This form is mainly observed in immunocompromised individuals, particularly those with HIV infection [7–9]. In this article, we report a case of hereditary EV, complicated by multiple squamous cell carcinomas, in a 19-year-old male with no evidence of consanguinity or involvement of other family members. Although the majority of EV cases are autosomal recessive, cases of cross-heredity and sporadic mutations have also been documented in the literature. The familial form of EV is the most frequent and common [9]. In our case, however, there was no family history of EV, nor any notion of consanguinity. It is therefore possible that our patient's case was sporadic. EV is a multifactorial disease, dependent on several factors, notably genetic, immunological, and environmental (actinic). These different factors play different roles in the clinical manifestations and complications of the disease [2]. It has been reported that certain types of HPV, notably HPV 5 and 8, are the most frequently found in patients developing cutaneous cancers, in over 90% of cases [10]. The majority of cases of cutaneous cancer in EV occur between the ages of 30 and 40 [3, 11]. The fact that the scalp is a particularly photo-exposed area suggests that high sun exposure in the tropics, as in Togo, may have played a decisive role in the early onset of carcinoma in our patient. The high solar gradient in Africa and its impact on the epidemiological profile of skin cancers in other genodermatoses such as albinism and xeroderma pigmentosum have been reported in numerous studies [3, 12, 13].

This clinical case also raises the challenges associated with the management and follow-up of genodermatoses such as albinism, xeroderma pigmentosum and EV in sub-Saharan Africa. Indeed, the populations are confronted with socioeconomic problems and lack of access to quality care, making access to preventive measures such as sun creams and therapeutic measures such as anticancer drugs difficult [14]. In our particular case, the patient has no financial means to carry out extension and pretherapeutic check-ups, nor for systemic anticancer treatment. The prognosis for this patient is therefore poor, in our context of lack of social security and effective universal health coverage.

## 4. Conclusion

We report a case of EV complicated by multiple cutaneous carcinomas of the scalp in a 19-year-old subject. The particularity of this case lies in the early onset of multiple cutaneous carcinomas. This case also presents a therapeutic and prognostic interest in view of the numerous obstacles, notably the number and size of lesions and the patient's economic difficulties. In sub-Saharan Africa, characterized by fragile health systems and high solar gradients, particular attention must be paid to the diagnosis and follow-up of patients with genodermatoses at risk of carcinomatous degeneration, such as EV.
